# Mesenchymal Stem Cells: A New Choice for Nonsurgical Treatment of OA? Results from a Bayesian Network Meta-Analysis

**DOI:** 10.1155/2021/6663003

**Published:** 2021-02-02

**Authors:** Ziqin Cao, Yajia Li, Fuqiang Gao, Ren Wu, Pengcheng Dou, Wanchun Wang, Qiangxiang Li

**Affiliations:** ^1^Department of Orthopedics, The Second Xiangya Hospital, Central South University, Changsha, Hunan 410011, China; ^2^Ningxia Geriatric Disease Clinical Research Center, People's Hospital of Ningxia Hui Autonomous Region, Yinchuan, Ningxia Hui Autonomous Region 750001, China; ^3^Department of Dermatology, Xiangya Hospital, Central South University, Changsha, China; ^4^Department of Orthopedics, China-Japan Friendship Hospital, China-Japan Friendship Institute of Clinical Medicine, Chinese Academy of Medical Sciences, Peking Union Medical College, Graduate School of Peking Union Medical College, Beijing, China; ^5^National Clinical Research Center for Geriatric Disorders of Xiangya Hospital, Central South University (Sub-Center of Ningxia), Yinchuan, Ningxia Hui Autonomous Region 750001, China; ^6^Hunan People's Hospital, Department of Hunan Institute of Geriatrics, Changsha 410002, China

## Abstract

**Objective:**

Osteoarthritis (OA) is the most common degenerative joint disease, causing joint pain, stiffness, and even disability. Guidelines recommend intra-articular injections as an alternative treatment to relieve OA symptoms for patients who demonstrate poor tolerability or compliance to oral administration of drugs. Mesenchymal stem cells (MSCs) are a potential treatment for of OA. We conducted this network meta-analysis to comprehensively compare the efficacy and safety between hyaluronic acid (HA), corticosteroids (GCs), platelet-rich plasma (PRP), and MSCs.

**Design:**

Systematic review and Bayesian network meta-analysis. *Data Sources*. Relevant studies, published from January 2000 to January 2020, in the PubMed, Cochrane library, EMBASE, and CKNI databases.

**Methods:**

Bayesian network and conventional meta-analyses were conducted. Pain relief, functional improvement, improvement in joint stiffness, and risk of adverse effects (AEs) were assessed.

**Results:**

Twenty-five articles with 4642 patients were included. Overall, MSC therapy was the most effective treatment for pain relief (standardized mean difference compared with placebo = 3.61, 95% CI [1.87 to 5.35]). Both MSC and PRP therapies improved every symptom of OA effectively and have an advantage over HA and GCs which are recommended by guidelines. MSCs, PRP, HA, and GCs are tolerated well for patients in long-term treatment of OA compared with placebo.

**Conclusions:**

The results show that MSCs relieve pain, stiffness, and dysfunction due to OA better than PRP, HA, and GCs and are not statistically correlated with greater safety concerns. More high-quality trials are needed to reconfirm the findings of this study, however, standardization of preparation of MSCs and PRP should be investigated in the future.

## 1. Introduction

Osteoarthritis (OA) is a common joint disease characterized by cartilage defects, joint space narrowing, and osteophyte formation. It mainly affects weight-bearing joints such as the ankle, knee, and hip joints. It often leads to local pain and stiffness in the early stage and to physical disability and loss of function in the later stages. Approximately 302 million people are affected by OA worldwide every year [1].

Nonsteroidal anti-inflammatory drugs (NSAIDs), tramadol and duloxetine, which are usually recommended by most guidelines such as those of the American College of Rheumatology/Arthritis Foundation Guideline (ACR), are regarded as classical drugs for nonsurgical treatment of OA [1]. However, these drugs evoke some tolerability and safety concerns [2] for the reported gastrointestinal and cardiovascular adverse events caused by the long-term use of them [3]. Intra-articular injection with hyaluronic acid (HA) or corticosteroids (GCs), which are recommended conditionally by OARSI guidelines [4], serves as an alternative approach to relieve OA symptoms for patients who demonstrate poor tolerability or compliance to oral administration of drugs. Recently, some novel intra-articular agents have been adopted to treat OA, including platelet-rich plasma (PRP) and mesenchymal stem cells (MSCs). A clinical study with a 30-month follow-up period found that patients received MSC treatments exhibiting significant therapeutic benefits without severe adverse events (AEs) [5], while a randomized clinical trial (RCT) has reported higher efficacy and safety for PRP therapy compared with other treatments [6]. Many meta-analyses and systematic reviews have already investigated the efficacy and safety of different intra-articular injection therapies for OA. Zhao et al. [7] conducted a network meta-analysis comparing clinical outcomes between four types of intra-articular injections (PRP, HA, CS, HA, and PRP) on hip OA management and reported that PRP can significantly relieve the pain related to OA. However, some limitations exist in their study design. Foremost, they only compared the pain-scale value at 1, 3, and 6 months directly, ignoring the divergence among different outcome-scale baseline values of each group shown in the published papers. This may reduce the reliability of the results of their meta-analysis. To counteract this, we included RCTs that used mesenchymal stem cell injection in our literature retrieval and selected “the changes from baseline” as one of the outcome measures to reduce the biases related to heterogeneity of baseline values. To deepen the analysis of the effects of the four types of intra-articular therapies (MSCs, PRP, HA and GC), we compared the values of several additional efficacy endpoints, including pain relief, function, and reduction in stiffness, among the groups.

## 2. Method

### 2.1. Literature Search

PubMed, Cochrane library, EMBASE, and CKNI databases from January 2000 to January 2020 were systematically searched. The retrieval strategy consisted of ((“mesenchymal stem cell” OR “mesenchymal stromal cell” OR “MSC”) OR (“hyaluronic acid” OR “Hyaluronan” OR “Sodium Hyaluronate” OR “HA”) OR (“platelet rich plasma” OR “platelet-rich plasma” OR “PRP”) OR (“glucocorticoid” OR “corticosteroid” OR “GC”) AND (“degenerative joint disease” OR “osteoarthritis” OR “OA”)) using “randomized controlled trial” as a filter.

We also screened the reference lists of previous studies to identify potentially eligible articles. Articles were not restricted because of language.

### 2.2. Inclusion/Exclusion Criteria

Studies that met the following criteria were included: (1) prospective parallel-group RCTs; (2) studies involving at least two or more therapies using MSCs, PRP, HA, GCs, and placebo; (3) participants with OA of the hand, ankle, hip, or knee joint; and (4) studies reporting the changes from baseline of the outcome indicators.

The exclusion criteria were as follows: (1) single-arm studies with a dose-escalation design; (2) participants with temporomandibular joint OA; (3) observational studies, cross-over studies, conference abstracts, reviews, letters, or case reports; (4) pharmacodynamic and pharmacokinetic studies; and (5) any nonhuman experimental studies.

Two authors independently completed the literature search. Any divergence of views was resolved by discussion.

### 2.3. Quality Assessment and Data Extraction

Two authors conducted the methodological quality and bias assessment of studies using the Cochrane risk of the bias assessment tool [8]. Six indices were evaluated and ranked as low, unclear, or high risk of bias. Sources of bias included sequence generation, allocation concealment, blinding, incomplete outcome data, selection outcome reporting, and other sources of bias. Funnel plots and Egger's tests were used to check the publication bias for this network meta-analysis. Any network with an asymmetric funnel plot or with a *P* value of the Egger's test < 0.05 was considered to have significant publication bias. Two other authors reviewed the full manuscripts of all eligible studies and extracted relevant data from the studies, including author, publication year, number of patients enrolled, average age, sex ratio, joints affected, whether funded or not, intervention methods, mean follow-up, and outcome data. To minimize the impact of withdrawal bias, if possible, the intention-to-treat analysis data was preferred.

All disputes were resolved through discussion.

### 2.4. Outcome Measures

In light of the differences among the baseline values of each included study that may reduce the reliability of the results, we used the change from baseline to the last follow-up value (mean ± SD) to evaluate efficacy. The primary efficacy endpoints included pain relief and functional improvement. Reduced joint stiffness was the secondary efficacy endpoint. There was no restriction on the type of questionnaire used in pain assessment. The functional and stiffness subscales of the Western Ontario and McMaster University arthritis index (WOMAC) were used to evaluate the improvement of function and stiffness. If WOMAC scales were not measured or reported, any other functional or stiffness measurement scales that were used were adopted, such as the Lequesne index. The standardized mean difference (SMD) with 95% confidence intervals (CI) was used to eliminate the bias caused by results that used different scales. Safety endpoints included withdrawal due to adverse events and serious adverse events/deaths due to adverse reactions. The odds ratio (OR) with 95% CI was used to measure the relative safety of treatments against each other.

### 2.5. Statistical Analysis

We followed the methods of Cao et al. [8]. Stata/MP (version 13.0, Stata Corp, College Station, Texas, USA) was used to conduct a direct pairwise meta-analysis to assess the relative efficacy and safety of the treatments compared to placebo. *Q* and *I*^2^ statistics were used to evaluate heterogeneity across studies, with *I*^2^ > 50% or *P* < 0.05 indicating significant heterogeneity. If significant heterogeneity was found, the random effects model was preferred; otherwise, the fixed effects model was used.

The Bayesian network meta-analysis adopted the random effects model, using Stata/MP (version 13.0) and the Aggregated Data Drug Information System (ADDIS, version 1.16.8). With the Bayesian method, the number of studies in each comparison could be increased, the width of the CI could be narrowed, and the results would be more reliable [9–12]. In this study, a noninformation uniform normal prior distribution was used. Then, four different sets of starting values were fixed to fit the model, and 20,000 iterations (5000 times per chain) were yielded to obtain the posterior distribution of model parameters [13, 14]. The thinning interval was set to 10 for each chain and the burn-ins at 1000. The convergence of the iteration was evaluated using the Gelman-Rubin-Brooks method [15]. The consistency of the network element analysis was reconfirmed through global inconsistency and node split tests.

The posterior distribution medians generated the SMDs and ORs with 95% CI. Significant differences between treatments were considered when the corresponding 95% CI excluded one for OR or zero for SMD. *P* < 0.05 was considered statistically significant. The efficacy and safety of different treatments were ranked by the surface cumulative ranking method (SUCRA). To select the most effective drugs for two or more end points at the same time, cluster-ranking plots were created. Two subgroup analyses were conducted: according to the affected joint (hip, knee, hand, or ankle) and according to the length of follow-up.

## 3. Results

### 3.1. Study Selection

**Figure 1:**
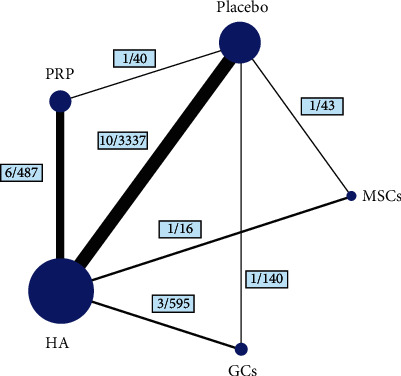
Structure of network formed by interventions. The lines between treatment nodes indicate the direct comparisons made within randomized controlled trials. Numbers (*n*/*n*) near the line indicate “number of trials/number of participants” of the related comparisons.

This study was conducted in accordance with the Preferred Reporting Items for Systematic Reviews and Meta-Analyses guidelines (PRISMA) [16]. Twenty-five articles were finally selected [17–41]. The selection criteria are shown in Figure S1. Five treatment groups (MSCs, PRP, HA, GCs, and placebo) were compared (Figure 1).

### 3.2. Study Characteristics

The analysis included 4642 patients. Only one study with 88 patients reported hand OA, one with 28 patients reported ankle OA, and three with 754 patients reported hip OA among the 25 eligible articles.

Across all included trials, the average age of patients was 59.80 years (range: 50.26-72.43 years), the proportion of male patients was 31.28% (range: 12.50%-89.29%), and the median follow-up time was 182 days (interquartile distance 180-336 days). The numbers of participants involved in each treatment group were 381 (GCs), 1477 (HA), 53 (MSCs), 327 (PRP), and 1038 (placebo). Specific baseline characteristics are shown in Table S1. The bias risk and methodological quality of all included studies were evaluated (Table S2). According to the result, the main types of bias are attrition bias, selection bias, and performance bias. Publication bias was found only in the networks for function and serious AEs (the *P* value of Egger's test > 0.05) (Figure S2).

### 3.3. Direct Meta-Analysis

The random effects model was adopted as significant heterogeneity was found for all endpoints. According to the results, MSCs were superior to placebo for pain relief (SMD 5.534, 95% CI [4.193 to 6.874]), functional improvement (SMD 2.314, 95% CI [1.531 to 3.097]), and reduction in stiffness (SMD 0.702, 95% CI [0.081 to 1.323]). PRP and HA demonstrated no long-term effect for any efficacy endpoint. GCs, compared with placebo, resulted in significantly greater pain (SMD -2.055, 95% CI [-2.466 to -1.645]) and disability (SMD -0.622, 95% CI [-0.962 to -0.283]), but no significant difference in stiffness (SMD 0.240, 95% CI [-0.093 to 0.572]). No treatment was associated with a significant increase in the risk of any safety endpoint (Table S3).

### 3.4. Network Meta-Analysis

#### 3.4.1. Efficacy Endpoint

**Figure 2:**
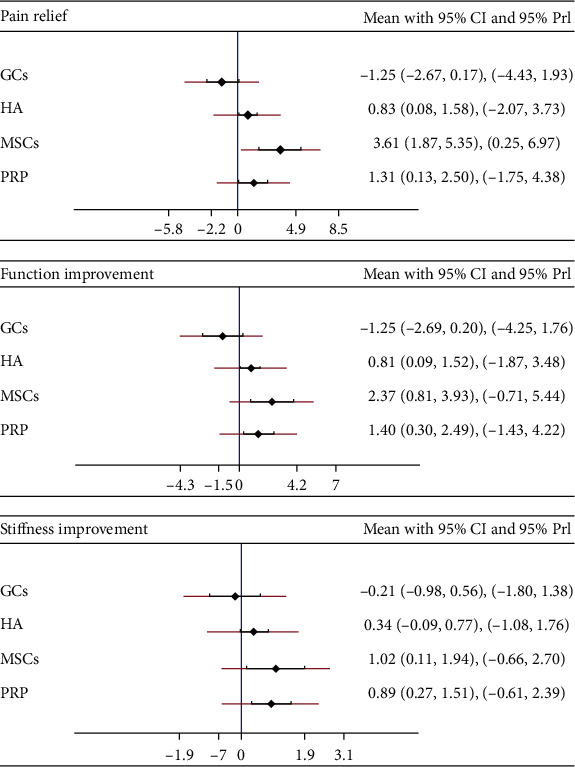
The forest plots of pain relief, function improvement, and stiffness improvement for network meta-analysis. SMD: standardized mean difference; CI: confidence interval; PrI: prediction interval.

No significant inconsistency was found in either global inconsistency tests or node-split tests for any of the three networks. The consistency model was adopted rather than the inconsistency model. The specific results are shown in Table 1 and Figure 2.

MSCs were the most effective treatment for pain relief (SMD compared with placebo 3.61, 95% CI [1.87 to 5.35]), and GCs were the least effective (SMD -1.25, 95% CI [-2.67 to 0.17]), showing no benefit compared with placebo. According to the SUCRA, MSCs were the most effective (SUCRA = 97.9%), followed by PRP (71.8%) and GCs (1.5%).

For the pain-relief network, MSCs were associated with the greatest functional improvement (SMD 2.37, 95% CI [0.81 to 3.93]). GCs were not different from placebo in long-term functional improvement (SMD-1.25, 95% CI [-2.69 to 0.20]). The most effective treatment for functional improvement based on SUCRA was MSCs, and the least effective was GCs (SUCRA for MSCs is 95.9% and for GCs 1.1%).

MSCs (SMD 1.02, 95% CI [0.11 to 1.94]) and PRP (SMD 0.89, 95% CI [0.27 to 1.51]) both showed superior effects compared to placebo for the secondary endpoint of reduced joint stiffness. HA (SMD 0.34, 95% CI [-0.09 to 0.77]) and GCs (SMD -0.21, 95% CI [-0.98 to 0.56]) had no benefit compared with placebo. Based on SUCRA, MSCs were the most effective treatment (SUCRA = 87.3%), followed by PRP (SUCRA = 84.4%). GCs were the least effective (SUCRA = 9.5%).

Cluster-rank plots are shown in Figure S3. MSCs were the optimum treatment for the combination of pain relief, function, and reduced stiffness.

The league plots comparing improvement in the primary endpoints between different treatments are presented in Table S4. The league plot of the secondary efficacy endpoint is presented in Table S5.

#### 3.4.2. Safety Endpoint

The consistency model was preferred because no inconsistency was reported in node-split tests or global inconsistency tests.

Treatments did not differ significantly in the proportion of withdrawals due to AEs or higher incidence of serious AEs or death due to AEs. However, according to SUCRA (Table 2), HA had the lowest withdrawal rate due to AEs (SUCRA = 62.2%), and GCs had the lowest rate of serious AEs or death due to AEs (SUCRA = 75.0%). The relative safety among treatments is shown in Table S6.

#### 3.4.3. Subgroup Analysis

The first subgroup analyses were performed to exploit the influence of length of follow-up. The median follow-up was 182 days, and only three studies had relatively short-term follow-up periods (42 days, 84 days, and 90 days). After excluding these studies, no substantial change was revealed compared with the results from the network analyses. (Table S7).

The second subgroup analysis was conducted to exploit the impacts from different affected joints, but only five of the 25 trials included in our study investigated joints other than the knee. Therefore, we only performed a subgroup analysis of patients with knee OA. This subgroup analysis did not identify any substantial change (Table S8).

## 4. Discussion

This is the first network meta-analysis using the Bayesian method simultaneously comparing the efficacy and safety of MSCs, PRP, HA, and GCs. As mentioned above, many systematic reviews [42, 43] have evaluated the efficacy of different intra-articular injection agents in the treatment of osteoarthritis, but most of these studies incorrectly took scale scores at different follow-up stages as outcome measures, ignoring the heterogeneities between the baseline scores of cohorts in different trials and how they affect the results. This is the reason we used the changes from baseline scores as the outcome measures in this study. The limited number of eligible articles in this meta-analysis could decrease the reliability, validity, and power of the results. In view of this, the Bayesian method was adopted to increase the number of studies within each comparison. The main findings are as follows: (1) MSC therapy demonstrates the greatest efficacy for pain relief for OA with significant superiority over PRP, HA, and GCs; (2) both MSCs and PRP effectively can improve various symptoms of OA and are better than HA and the GCs recommended by the guidelines; (3) GCs are not suitable for long-term intra-articular administration for OA because they do not provide relief from joint stiffness and can cause greater pain and dysfunction; meanwhile, MSCs, PRP, and HA all are well tolerated and effective for long-term treatment of OA patients.

There are some limitations to this study. First of all, compared with traditional pairwise meta-analysis, network meta-analysis has more confounding factors and difficulties to handle, and the reliability of its results is largely related to the number of studies included. However only RCTs with prospective parallel-group design are incorporated into analysis to avoid unmanageable confounding factors existing in cross-over RCTs and observational studies. Nevertheless, cross-over RCTs and especially observational studies play an indispensable role in assessing the long-term effectiveness and safety of therapy. This may be one of the reasons for the small number of studies included in this analysis. Second, only the changes from the baseline score at the last follow-up were collected and analyzed. Hence, the dynamic changes of the outcomes during the entire follow-up period are not reflected in the analysis, which prevents the evaluation of the early and mid-term effects and safety of different treatments. Therefore, publication bias may be a potential problem. Funnel plots and Egger's tests were performed in this study, and publication bias was only found in networks for function and serious AEs. These results should be interpreted cautiously. Third, the number of included cases subjected to MSC intra-articular injection is small when compared with those of HA or placebo groups. This may reduce the reliability of the results and conclusions. More high-quality trials with eligible outcome measures are needed. Fourthly, SUCRA is widely used to rank the effects of each treatment and identify the best one. However, it ignores whether the difference between treatments is clinically meaningful. While one treatment may be rated as the best, the absolute difference between the best treatment and others may be trivial. The results of SUCRA should be interpreted with caution [44]. Finally, network meta-analysis is a particularly effective and powerful statistical method, but its stability and reliability are based on strict uniform standards. All included studies should be of sufficient homogeneity with similar subject eligibility criteria, outcome measures, and treatment standardization. The limited homogeneity, partially attributed to the nonunified preparation procedures of MSCs and PRP, is an inherent limitation in the body of evidence in this area that must be acknowledged. Standardized definitions of preparation, as well as surgical technique, are required to improve the quality of evidence and its credibility.

## 5. Conclusion

Twenty-five studies assessing 4642 patients were analyzed in this work. The results show that intra-articular administration of MSCs can significantly relieve joint pain, reduce stiffness, and improve physical function in OA patients compared with PRP, HA and GC treatments, and is not statistically correlated with greater safety concerns. GCs are not suitable for the long-term treatment of OA. More high-quality trials are needed to reconfirm the findings of this study. Standardization of preparation of MSCs and PRP should be developed in the future.

## Figures and Tables

**Table 1:** The details of SURCA and mean rank of efficacy endpoints.

Treatment	SMD (95% CI) for pain relief	SURCA for pain relief (%)	Mean rank for pain relief	SMD (95% CI) for function improvement	SURCA for function improvement (%)	Mean rank for function improvement	SMD (95% CI) for stiffness improvement	SURCA for stiffness improvement (%)	Mean rank for stiffness improvement
Placebo	Reference	24.3	4.0	Reference	24.5	4.0	Reference	19.8	4.2
MSCs	3.61 (1.87, 5.35)	97.9	1.1	2.37 (0.81, 3.93)	95.9	1.2	1.02 (0.11, 1.94)	87.6	1.5
PRP	1.31 (0.13, 2.50)	71.8	2.1	1.40 (0.30, 2.49)	75.9	2.0	0.89 (0.27, 1.51)	84.1	1.6
HA	0.83 (0.08, 1.58)	54.5	2.8	0.81 (0.09, 1.52)	52.7	2.9	0.34 (-0.09, 0.77)	48.7	3.1
GCs	-1.25 (-2.67, 0.17)	1.5	4.9	-1.25 (-2.69, 0.20)	1.1	5.0	-0.21 (-0.98, 0.56)	9.7	4.6

**Table 2:** The details of SURCA and mean rank of safety endpoints.

Treatment	OR (95% CI) for withdrawal due to AEs	SURCA for withdrawal due to AEs (%)	Mean rank for withdrawal due to AEs	OR (95% CI) for serious AEs or death	SURCA for serious AEs or death (%)	Mean rank for serious AEs or death	OR (95% CI) for injection site discomfort	SURCA for injection site discomfort (%)	Mean rank for injection site discomfort
Placebo	Reference	57.8	2.7	Reference	60.8	2.6	Reference	86.0	1.6
MSCs	-0.15 (-4.22, 3.93)	56.3	2.7	0.20 (-1.70, 2.10)	42.7	3.3	0.31 (-2.07, 2.68)	66.5	2.3
PRP	0.14 (-1.43, 1.70)	48.0	3.1	1.24 (0.29, 2.19)	39.1	3.4	0.34 (-1.02, 1.70)	11.8	4.5
HA	-0.05 (-0.74, 0.64)	62.2	2.5	0.89 (0.30, 1.47)	32.4	3.7	0.40 (-0.43, 1.23)	32.6	3.7
GCs	0.50 (-0.64, 1.63)	25.8	4.0	0.55 (-0.49, 1.59)	75.0	2.0	-0.54 (-2.58, 1.49)	53.2	2.9

## Data Availability

The data used to support the findings of this study are included within the article and supplementary appendix documents.

## Supplementary Materials

Supplementary 1 Figure S1: PRISMA flow diagram.

Supplementary 2 Figure S2: publication bias and Egger test plots.

Supplementary 3 Figure S3: cluster-rank plots.

Supplementary 4 Table S1: baseline characteristics of included studies.

Supplementary 5 Table S2: methodological quality and risk of bias evaluation.

Supplementary 6 Table S3: the details of direct meta-analysis.

Supplementary 7 Table S4: the detailed results of network meta-analysis for pain (red) and function (blue) (data are standardized mean difference, from the top left to the bottom right, higher comparator vs. lower comparator, and their related 95% CI).

Supplementary 8 Table S5: the detailed results of network meta-analysis for stiffness (data are standardized mean difference, from the top left to the bottom right, higher comparator vs. lower comparator, and their related 95% CI).

Supplementary 9 Table S6: the detailed results of network meta-analysis for withdrawal due to AEs (red) and serious AEs or death (blue) (data are standardized mean difference, from the top left to the bottom right, higher comparator vs. lower comparator, and their related 95% CI).

Supplementary 10 Table S7: the details of results for subgroup analysis of long-term follow-up.

Supplementary 11 Table S8: the details of results for subgroup analysis of knee OA.
