# Supplementary figures and images for: Mesenchymal Stem Cells: A New Choice for Nonsurgical Treatment of OA? Results from a Bayesian Network Meta-Analysis
Source: Biomed Res Int. 2021 Feb 2;2021:6663003. doi: 10.1155/2021/6663003 (PMC7876826; doi:10.1155/2021/6663003)

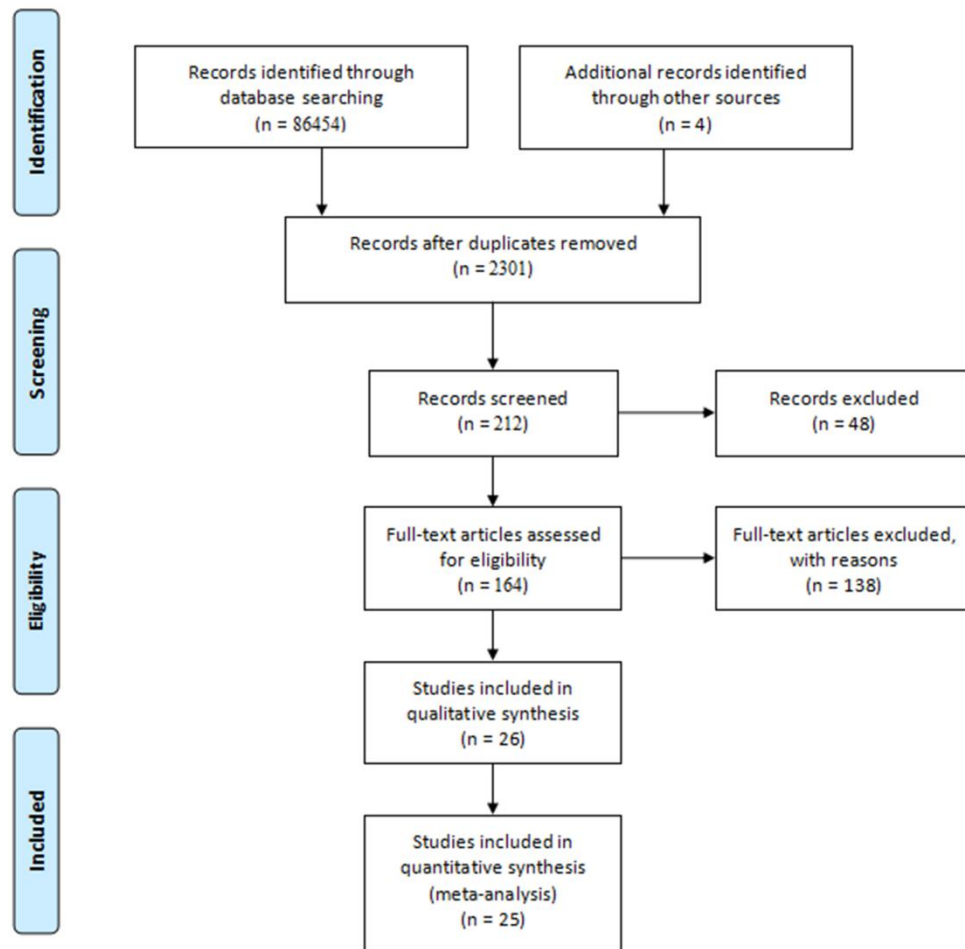

**Figure S1.** PRISMA Flow Diagram.

Supplement: Supplementary 1 — Figure S1: PRISMA flow diagram. [file 6663003.f1.pdf]
