# Supplementary material for: Mesenchymal Stem Cells: A New Choice for Nonsurgical Treatment of OA? Results from a Bayesian Network Meta-Analysis
Source: Biomed Res Int. 2021 Feb 2;2021:6663003. doi: 10.1155/2021/6663003 (PMC7876826; doi:10.1155/2021/6663003)
Supplement: Supplementary 2 — Figure S2: publication bias and Egger test plots. [file 6663003.f2.pdf]

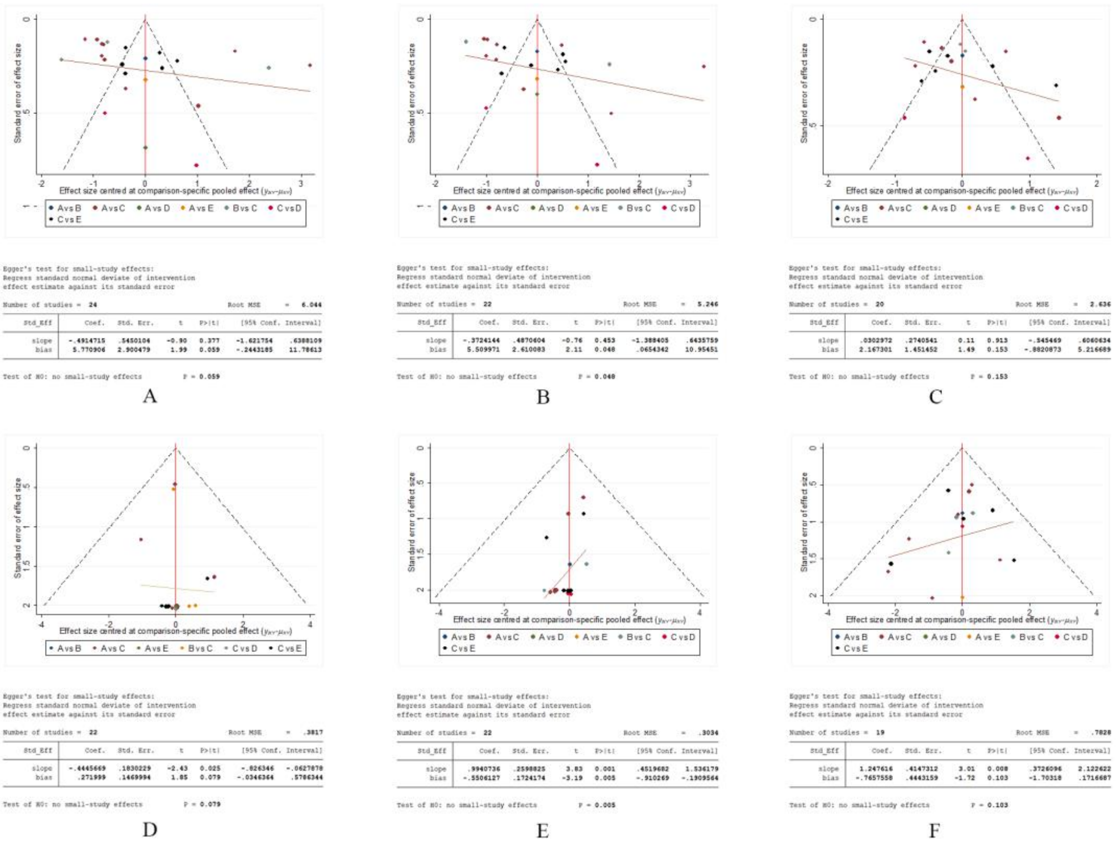

Figure S2. Publication bias and Egger test plots. (A) Pain relief. (B) Function improvement (C) Stiffness improvement. (D) Withdrawal due to AEs. (E) Serious AEs or death. (F) Injection site discomfort.
