# Supplementary material for: Mesenchymal Stem Cells: A New Choice for Nonsurgical Treatment of OA? Results from a Bayesian Network Meta-Analysis
Source: Biomed Res Int. 2021 Feb 2;2021:6663003. doi: 10.1155/2021/6663003 (PMC7876826; doi:10.1155/2021/6663003)
Supplement: Supplementary 3 — Figure S3: cluster-rank plots. [file 6663003.f3.pdf]

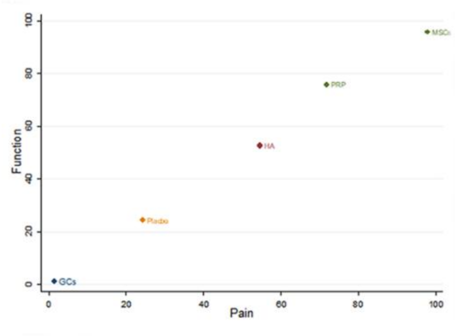

A

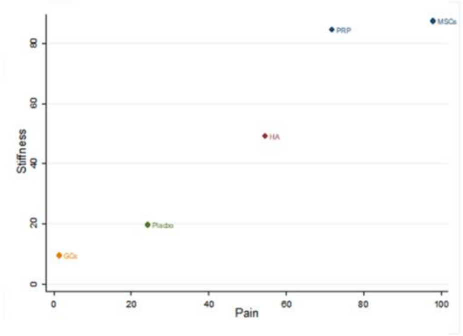

B

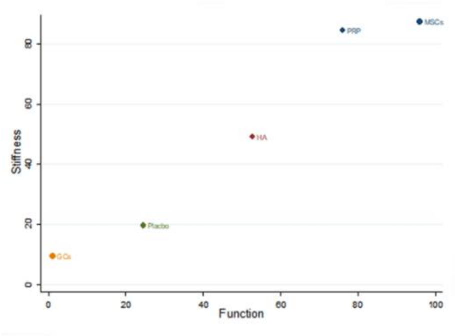

C

Figure S3. Cluset-rank plots. (A) The cluster-rank plot of pain relief and function improvement for different intraaticular injection treatments. (B) The cluster-rank plot of pain relief and stiffness improvement for different intraaticular injection treatments. (C) The cluster-rank plot of function improvement and stiffness improvement for different intraaticular injection treatments. (The cluster-rank value is the product of the abscissa and ordinate of each treatment).
