# Supplementary material for: Mesenchymal Stem Cells: A New Choice for Nonsurgical Treatment of OA? Results from a Bayesian Network Meta-Analysis
Source: Biomed Res Int. 2021 Feb 2;2021:6663003. doi: 10.1155/2021/6663003 (PMC7876826; doi:10.1155/2021/6663003)
Supplement: Supplementary 4 — Table S1: baseline characteristics of included studies. [file 6663003.f4.pdf]

Table S1. Baseline Characteristics of included Studies

Author	No.	Year	Number of patients	Mean age	Gender ratio (Male/Female)	Target joint	Funded or Sponsored	Experimental intervention	Control intervention	Follow-up period
Petrella RJ et al.[17]	1	2006	105	63.13	58/47	Knee	Yes	HA	Placebo	84days
Sánchez M et al.[18]	2	2012	176	59.71	80/96	Knee	No	PRP	HA	180days
Arden NK et al.[19]	3	2014	2108	62.68	108/110	Knee	No	HA	Placebo	42days
Neustadt D et al.[20]	4	2020	336	58.8	175/161	Knee	No	HA	Placebo	196days
Trueba Davalillo CÁ et al.[21]	5	2015	195	62.75	82/113	Knee	No	HA	GCs	360days
Monfort J et al.[22]	6	2015	88	62.8	11/77	Hand	No	HA	GCs	180days
Vaquerizo V et al.[23]	7	2013	96	63.6	38/58	Knee	Yes	PRP	HA	336days
Buendía-López D et al.[24]	8	2018	98	56.73	47/51	Knee	No	PRP	HA	360days
Richette P et al.[25]	9	2009	85	60.14	35/50	Hip	Yes	HA	Placebo	90days
McAlindon TE et al.[26]	10	2020	140	58.15	65/75	Knee	Yes	GCs	Placebo	730days

Wu YT et al.[27]	11	2018	40	63.25	10/30	Knee	No	PRP	Placebo	180days
Altman RD et al.[28]	12	2004	346	63.1	156/190	Knee	Yes	HA	Placebo	182days
Raeissadat SA et al.[29]	13	2017	69	58.2	13/56	Knee	No	PRP	HA	180days
Brander V et al.[30]	14	2019	357	60.3	146/211	Hip	Yes	HA	Placebo	182days
Louis ML et al.[31]	15	2017	48	50.85	25/23	Knee	Yes	PRP	HA	180days
Spitzer Al et al.[32]	16	2010	312	59	151/161	Hip	Yes	HA	GCs	182days
Emadedin M et al et al.[33]	17	2018	43	53.37	27/16	Knee	Yes	MSCs	Placebo	180days
Zheping H et al.[34]	18	2018	16	52.0	3/13	Knee	Yes	MSCs	HA	365days
Bastos R et al.[35]	19	2019	47	57.29	24/23	Knee	No	MSCs	GCs	360days
Teng-Le Hu et al.[36]	20	2011	200	65.0	48/152	Knee	No	HA	Placebo	150days
Seyed Ahmad Raeissadat et al.[37]	21	2014	139	58.76	23/116	Knee	No	PRP	HA	312days
Matas J et al.[38]	22	2018	29	55.90	13/16	Knee	No	MSCs	HA	360days

Cohen MM et al.[39]	23	2008	28	50.26	25/3	Ankle	Yes	HA	Placebo	180days
Chevalier X et al.[40]	24	2009	251	63.54	73/178	Knee	Yes	HA	Placebo	182days
DeCaria JE et al.[41]	25	2011	30	72.43	16/14	Knee	No	HA	Placebo	180days
