# Supplementary material for: Mesenchymal Stem Cells: A New Choice for Nonsurgical Treatment of OA? Results from a Bayesian Network Meta-Analysis
Source: Biomed Res Int. 2021 Feb 2;2021:6663003. doi: 10.1155/2021/6663003 (PMC7876826; doi:10.1155/2021/6663003)
Supplement: Supplementary 5 — Table S2: methodological quality and risk of bias evaluation. [file 6663003.f5.pdf]

Table S2. Methodological quality and risk of bias evaluation.

Article	1.Sequence generation	2.Allocation concealment	3.Blinding	4.Incomplete outcome data	5.Selective outcome reporting	6.Other source of bias
Petrella RJ et al.[17]	High risk of bias	Low risk of bias	Low risk of bias	High risk of bias	Low risk of bias	Unclear risk of bias
Sánchez M et al.[18]	Low risk of bias	Low risk of bias	Low risk of bias	Low risk of bias	Low risk of bias	Unclear risk of bias
Arden NK et al.[19]	High risk of bias	High risk of bias	Low risk of bias	Low risk of bias	Low risk of bias	Unclear risk of bias
Neustadt D et al.[20]	Low risk of bias	Low risk of bias	Low risk of bias	Low risk of bias	Low risk of bias	Unclear risk of bias
Trueba Davalillo CÁ et al.[21]	Unclear risk of bias	Low risk of bias	Low risk of bias	Low risk of bias	Low risk of bias	Unclear risk of bias
Monfort J et al.[22]	Low risk of bias	Low risk of bias	Low risk of bias	High risk of bias	Low risk of bias	Unclear risk of bias
Vaquerizo V et al.[23]	Low risk of bias	Low risk of bias	Low risk of bias	High risk of bias	Low risk of bias	Unclear risk of bias
Buendía-López D et al.[24]	Low risk of bias	Unclear risk of bias	Low risk of bias	Low risk of bias	Low risk of bias	Unclear risk of bias

Richette P et al.[25]	Low risk of bias	Low risk of bias	Low risk of bias	Low risk of bias	Low risk of bias	Unclear risk of bias
McAlindon TE et al.[26]	Low risk of bias	Low risk of bias	Low risk of bias	Low risk of bias	Low risk of bias	Unclear risk of bias
Wu YT et al.[27]	High risk of bias	High risk of bias	Low risk of bias	Low risk of bias	Low risk of bias	Unclear risk of bias
Altman RD et al.[28]	Low risk of bias	Low risk of bias	Low risk of bias	Low risk of bias	Low risk of bias	Unclear risk of bias
Raeissadat SA et al.[29]	Low risk of bias	Low risk of bias	Low risk of bias	Low risk of bias	Low risk of bias	Unclear risk of bias
Brander V et al.[30]	Unclear risk of bias	High risk of bias	Low risk of bias	Low risk of bias	Low risk of bias	Unclear risk of bias
Louis ML et al.[31]	Low risk of bias	Low risk of bias	Low risk of bias	Low risk of bias	Low risk of bias	Unclear risk of bias
Spitzer Al et al.[32]	Low risk of bias	Low risk of bias	Low risk of bias	Low risk of bias	Low risk of bias	Unclear risk of bias
Emadedin M et al.[33]	Low risk of bias	Low risk of bias	Low risk of bias	Low risk of bias	Low risk of bias	Unclear risk of bias

Zheping H et al.[34]	Low risk of bias	High risk of bias	Low risk of bias	Low risk of bias	Low risk of bias	Unclear risk of bias
Bastos R et al.[35]	Low risk of bias	Low risk of bias	Low risk of bias	Low risk of bias	Low risk of bias	Unclear risk of bias
Teng-Le Hu et al.[36]	High risk of bias	High risk of bias	Low risk of bias	Low risk of bias	Low risk of bias	Unclear risk of bias
Seyed Ahmad Raeissadat et al.[37]	Low risk of bias	High risk of bias	Low risk of bias	Low risk of bias	Low risk of bias	Unclear risk of bias
Matas J et al.[38]	Low risk of bias	Low risk of bias	Low risk of bias	Low risk of bias	Low risk of bias	Unclear risk of bias
Cohen MM et al.[39]	High risk of bias	Low risk of bias	Low risk of bias	Low risk of bias	Low risk of bias	Unclear risk of bias
Chevalier X et al.[40]	Low risk of bias	Low risk of bias	Low risk of bias	Low risk of bias	Low risk of bias	Unclear risk of bias
DeCaria JE et al.[41]	Low risk of bias	Low risk of bias	Low risk of bias	Low risk of bias	Low risk of bias	Unclear risk of bias
