# Supplementary material for: Mesenchymal Stem Cells: A New Choice for Nonsurgical Treatment of OA? Results from a Bayesian Network Meta-Analysis
Source: Biomed Res Int. 2021 Feb 2;2021:6663003. doi: 10.1155/2021/6663003 (PMC7876826; doi:10.1155/2021/6663003)
Supplement: Supplementary 6 — Table S3: the details of direct meta-analysis. [file 6663003.f6.pdf]

Table S3. The details of direct meta-analysis.

Comparison	Target joint	SMD(95%CI) for Pain relief	SMD(95%CI) for Function improvement	SMD(95%CI) for Stiffness improvement	OR(95%CI) for Withdrawal due to AEs	OR(95%CI) for Serious AEs or death	OR(95%CI) for Injection site discomfort
GCs vs placebo	Knee	-2.055(-2.466,-1.645)	-0.622(-0.962,-0.283)	0.240(-0.093,0.572)	Insufficient comparision	Insufficient comparision	Insufficient comparision
HA vs placebo	Knee, hip, ankle	0.942(0.084,1.801)	0.865(0.138,1.592)	0.388(-0.062,0.838)	1.023(0.479,2.182)	0.498(0.168,1.477)	0.500(0.282,0.885)
MSCs vs placebo	Knee	5.534 (4.193,6.874)	2.314(1.531,3.097)	0.702(0.081,1.323)	Insufficient comparision	Insufficient comparision	Insufficient comparision
PRP vs placebo	Knee	0.571(-0.062,1.204)	0.347(-0.278,0.972)	0.345(-0.280,0.969)	Insufficient comparision	Insufficient comparision	Insufficient comparision
