# Supplementary material for: Mesenchymal Stem Cells: A New Choice for Nonsurgical Treatment of OA? Results from a Bayesian Network Meta-Analysis
Source: Biomed Res Int. 2021 Feb 2;2021:6663003. doi: 10.1155/2021/6663003 (PMC7876826; doi:10.1155/2021/6663003)
Supplement: Supplementary 7 — Table S4: the detailed results of network meta-analysis for pain (red) and function (blue) (data are standardized mean difference, from the top left to the bottom right, higher comparator vs. lower comparator, and their related 95% CI). [file 6663003.f7.pdf]

Table S4. The detailed results of network meta-analysis for pain (Red) and function (Blue) (Data are standardised mean difference, from the top left to the bottom right, higher comparator vs lower comparator, and their related 95% CI)

**MSCs**	-0.94 (-2.72 to 0.83)	-1.52 (-3.05 to 0.02)	-4.18 (-6.45 to -1.91)	-2.45 (-4.05 to -0.85)
2.30 (0.36 to 4.24)	**PRP**	-0.57 (-1.49 to 0.35)	-3.24 (-5.19 to -1.28)	-1.51 (-2.68 to -0.33)
2.78 (1.10 to 4.46)	0.48 (-0.51 to 1.48)	**HA**	-2.66 (-4.42 to -0.91)	-0.93 (-1.79 to -0.08)
4.86 (2.75 to 6.97)	2.57 (0.93 to 4.20)	2.08 (0.77 to 3.40)	**GCs**	1.73 (-0.02 to 3.48)
3.61 (1.87 to 5.35)	1.31 (0.13 to 2.50)	0.83 (0.08 to 1.58)	-1.25 (-2.67 to 0.17)	**Placebo**
