# Supplementary material for: Mesenchymal Stem Cells: A New Choice for Nonsurgical Treatment of OA? Results from a Bayesian Network Meta-Analysis
Source: Biomed Res Int. 2021 Feb 2;2021:6663003. doi: 10.1155/2021/6663003 (PMC7876826; doi:10.1155/2021/6663003)
Supplement: Supplementary 8 — Table S5: the detailed results of network meta-analysis for stiffness (data are standardized mean difference, from the top left to the bottom right, higher comparator vs. lower comparator, and their related 95% CI). [file 6663003.f8.pdf]

Table S5. The detailed results of network meta-analysis for stiffness (Data are standardised mean difference, from the top left to the bottom right, higher comparator vs lower comparator, and their related 95% CI)

**MSCs**				
0.13 (-0.88 to 1.14)	**PRP**			
0.68 (-0.21 to 1.57)	0.55 (0.05 to 1.04)	**HA**		
1.24 (0.10 to 2.37)	1.10 (0.23 to 1.97)	0.55 (-0.17 to 1.28)	**GCs**	
1.02 (0.11 to 1.94)	0.89 (0.27 to 1.51)	0.34 (-0.09 to 0.77)	-0.21 (-0.98 to 0.56)	**Placebo**
