# Supplementary material for: Mesenchymal Stem Cells: A New Choice for Nonsurgical Treatment of OA? Results from a Bayesian Network Meta-Analysis
Source: Biomed Res Int. 2021 Feb 2;2021:6663003. doi: 10.1155/2021/6663003 (PMC7876826; doi:10.1155/2021/6663003)
Supplement: Supplementary 9 — Table S6: the detailed results of network meta-analysis for withdrawal due to AEs (red) and serious AEs or death (blue) (data are standardized mean difference, from the top left to the bottom right, higher comparator vs. lower comparator, and their related 95% CI). [file 6663003.f9.pdf]

**Table 6.** The detailed results of network meta-analysis for withdrawal due to AEs (Red) and serious AEs or death (Blue) (Data are standardised mean difference, from the top left to the bottom right, higher comparator vs lower comparator, and their related 95% CI).

| | | | | |
|---|---|---|---|---|
| **MSCs** | 0.03 (-2.55 to 2.61) | 0.09 (-2.24 to 2.42) | -0.85 (-3.89 to 2.19) | -0.31 (-2.68 to 2.07) |
| -0.28 (-4.55 to 3.99) | **PRP** | 0.06 (-1.07 to 1.19) | -0.88 (-3.16 to 1.40) | -0.34 (-1.70 to 1.02) |
| -0.10 (-4.12 to 3.92) | 0.18 (-1.26 to 1.63) | **HA** | -0.94 (-2.93 to 1.05) | -0.40 (-1.23 to 0.43) |
| -0.64 (-4.77 to 3.48) | -0.36 (-2.08 to 1.36) | -0.54 (-1.48 to 0.39) | **GCs** | 0.54 (-1.49 to 2.58) |
| -0.15 (-4.22 to 3.93) | 0.14 (-1.43 to 1.70) | -0.05 (-0.74 to 0.64) | 0.50 (-0.64 to 1.63) | **Placebo** |
