# Supplementary material for: Mesenchymal Stem Cells: A New Choice for Nonsurgical Treatment of OA? Results from a Bayesian Network Meta-Analysis
Source: Biomed Res Int. 2021 Feb 2;2021:6663003. doi: 10.1155/2021/6663003 (PMC7876826; doi:10.1155/2021/6663003)
Supplement: Supplementary 10 — Table S7: the details of results for subgroup analysis of long-term follow-up. [file 6663003.f10.pdf]

Table S7. The details of results for subgroup analysis of long-term follow-up.

Treatment	SMD (95% CI) for Pain relief	SURCA for Pain relief , %	SMD (95% CI) for Function improvement	SURCA for Function improvement, %	SMD (95% CI) for Stiffness improvement	SURCA for Stiffness improvement, %	OR (95% CI) for withdral due to AEs	SURCA for Withdrawal due to AEs, %	OR (95% CI) for Serious AEs or death	SURCA for Serious AEs or death, %	OR (95% CI) for Injection site discomfort	SURCA for Injection site discomfort, %
Placebo	Reference	23.1	Reference	22.9	Reference	15.2	Reference	50.9	Reference	62.1	Reference	81.5
MSCs	3.80 (1.96,5.63)	99.6	2.57 (0.93,4.21)	94.8	1.15 (0.17,2.13)	85.7	-0.27 (-4.36,3.81)	56.4	0.35 (-2.04,2.73)	42.7	0.07 (-1.88,2.01)	67.1
PRP	1.55 (0.25,2.85)	70.0	1.66 (0.46,2.86)	76.0	1.06 (0.34,1.78)	85.0	0.03 (-1.55,1.61)	49.7	0.39 (-1.00,1.78)	39.2	1.07 (-0.02,2.16)	13.1
HA	1.11 (0.20,2.01)	54.9	1.12 (0.24,1.99)	53.9	0.53 (-0.04,1.11)	51.0	-0.17 (-0.91,0.56)	65.8	0.46 (-0.44,1.35)	31.2	0.72 (-0.09,1.52)	34.7
GCs	-1.05 (-2.57,0.48)	2.4	-1.04 (-2.57,0.49)	2.4	-0.08 (-0.94,0.78)	13.1	0.38 (-0.78,1.54)	27.2	-0.51 (-2.55,1.54)	74.9	0.43 (-0.68,1.54)	53.6
