# Supplementary material for: Mesenchymal Stem Cells: A New Choice for Nonsurgical Treatment of OA? Results from a Bayesian Network Meta-Analysis
Source: Biomed Res Int. 2021 Feb 2;2021:6663003. doi: 10.1155/2021/6663003 (PMC7876826; doi:10.1155/2021/6663003)
Supplement: Supplementary 11 — Table S8: the details of results for subgroup analysis of knee OA. [file 6663003.f11.pdf]

Table 8. The details of results for subgroup analysis of knee OA.

Treatment	SMD (95% CI) for Pain relief	SURCA for Pain relief , %	SMD (95% CI) for Function improvement	SURCA for Function improvement, %	SMD (95% CI) for Stiffness improvement	SURCA for Stiffness improvement, %	OR (95% CI) for withdral due to AEs	SURCA for Withdrawal due to AEs, %	OR (95% CI) for Serious AEs or death	SURCA for Serious AEs or death, %	OR (95% CI) for Injection site discomfort	SURCA for Injection site discomfort, %
Placebo	Reference	25.2	Reference	25.0	Reference	20.0	Reference	50.9	Reference	60.4	Reference	83.9
MSCs	3.79 (2.12,5.45)	99.8	2.45 (0.85,4.05)	85.5	0.97 (0.09,1.86)	88.1	-0.10 (-4.28,4.09)	53.3	0.35 (-2.03,2.74)	41.7	0.26 (-1.68,2.20)	63.7
PRP	1.55 (0.39,2.71)	70.6	1.51 (0.33,2.68)	75.6	0.83 (0.21,1.44)	83.4	0.18 (-1.59,1.94)	43.2	0.40 (-0.98,1.79)	36.6	1.31 (0.24,2.37)	12.2
HA	1.11 (0.29,1.92)	54.4	0.93 (0.08,1.79)	53.0	0.27 (-0.19,0.74)	44.4	0.00 (-1.17,1.17)	51.9	0.47 (-0.41,1.34)	29.8	0.96 (0.19,1.73)	31.9
GCs	-2.56 (-4.33,-0.80)	0.1	-1.73 (-3.48,0.02)	0.7	-0.15 (-1.02,0.71)	14.2	-0.00 (-2.85,2.84)	50.7	-0.88 (-3.20,1.43)	81.5	0.48 (-0.80,1.76)	58.2
